# Seasonality in extra-pulmonary tuberculosis notifications in Germany 2004-2014- a time series analysis

**DOI:** 10.1186/s12889-021-10655-6

**Published:** 2021-04-06

**Authors:** Tanja Charles, Matthias Eckardt, Basel Karo, Walter Haas, Stefan Kröger

**Affiliations:** 1 Department for Infectious Disease Epidemiology, Robert Koch Institute, Berlin, Germany; 2 Postgraduate Training for Applied Epidemiology, Robert Koch Institute, Berlin, Germany; 3 European Programme for Intervention Epidemiology Training, ECDC, Solna, Sweden; 4 Centre for International Health Protection (ZIG), Robert Koch Institute, Berlin, Germany; 5 German Center for Infection Research (DZIF), partner site Hanover – Brunswick, Germany

**Keywords:** Tuberculosis, Germany, Seasonality, Time series, Infectious disease surveillance

## Abstract

**Background:**

Seasonality in tuberculosis (TB) has been found in different parts of the world, showing a peak in spring/summer and a trough in autumn/winter. The evidence is less clear which factors drive seasonality. It was our aim to identify and evaluate seasonality in the notifications of TB in Germany, additionally investigating the possible variance of seasonality by disease site, sex and age group.

**Methods:**

We conducted an integer-valued time series analysis using national surveillance data. We analysed the reported monthly numbers of started treatments between 2004 and 2014 for all notified TB cases and stratified by disease site, sex and age group.

**Results:**

We detected seasonality in the extra-pulmonary TB cases (*N* = 11,219), with peaks in late spring/summer and troughs in fall/winter. For all TB notifications together (*N* = 51,090) and for pulmonary TB only (*N* = 39,714) we did not find a distinct seasonality. Additional stratified analyses did not reveal any clear differences between age groups, the sexes, or between active and passive case finding.

**Conclusion:**

We found seasonality in extra-pulmonary TB only, indicating that seasonality of disease onset might be specific to the disease site. This could point towards differences in disease progression between the different clinical disease manifestations. Sex appears not to be an important driver of seasonality, whereas the role of age remains unclear as this could not be sufficiently investigated.

**Supplementary Information:**

The online version contains supplementary material available at 10.1186/s12889-021-10655-6.

## Background

Seasonality in tuberculosis (TB) has been described in studies in different parts of the world and among populations with varying TB incidences [1–19]. Most often, a seasonal peak was observed in spring/summer and a trough in autumn/winter. Development of disease is associated with immune status [16]. Therefore, general impaired immunity in winter could lead to an increase in disease onsets followed by a peak in diagnosis in spring/summer [17]. Furthermore, vitamin D deficiency in winter due to reduced sunlight exposure could enhance disease development [17], as vitamin D is believed to play an important protective role against infection and against mycobacteria. Another possible explanation for seasonality of TB is winter indoor crowding, as more time spent inside during the colder months could lead to increased transmission [8, 13], resulting in a peak in disease onsets and diagnoses some months later.

Understanding the mechanisms behind the seasonality of TB remains difficult, however, as the incubation period of TB and the delay between disease onset and diagnosis vary considerably [20]. At least two studies from the Netherlands showed that seasonality of TB varied by disease site: one found a seasonal peak in spring/summer in extra-pulmonary TB notifications only [16], while in another study the seasonality of the extra-pulmonary notifications seemed to drive the seasonal pattern found in TB notifications in non-natives [17]. However, many studies investigating seasonality in TB focus on all types of TB together, or on pulmonary TB only and do not discuss possible differences between extra-pulmonary and pulmonary TB. It could furthermore be expected that seasonality might depend on sex and age, as was found in at least two studies [6, 21], possibly due to the impact these factors could have on susceptibility for infection and disease progress [22–24].

It was our aim to evaluate if seasonality of TB can be detected in the cases notified in Germany between 2004 and 2014, additionally investigating the possible variance of seasonality by disease site, sex and age group. Germany is a country with a low TB incidence (6.7 per 100,000 population in 2017) [25]. To reach the WHO goal of “pre-elimination” of TB until 2035 (defined as < 1 TB case per 100,000), however, progress on reducing the number of new infections and early detection of active case is needed [26].

## Methods

### Data source

In this study we conducted a time series analysis using national notification data, which was obtained through the national electronic reporting system for surveillance of notifiable infectious diseases (SurvNet, implemented in 2001) [27] at the Robert Koch Institute, the German national public health institute (date of data extraction: 1st of March 2018). Ethical approval was not required for this study, as the analyses were performed on pseudo-anonymized notification data.

### Study design

We carried out a time series analysis to test our hypothesis that TB notifications show seasonality. In this study we included TB cases notified in Germany meeting the reference case definition (clinically diagnosed disease, clinically−/epidemiologically confirmed disease and clinically−/laboratory confirmed disease) [25] and with start of treatment between 2004 and 2014_._ To assess the seasonality we used the month and year of start of treatment as proxy for date of disease onset. We analyzed the data of one decade and did not include data after 2014, as the years 2015 and (to a lesser extent) 2016 were characterized by an unusual high immigration of persons seeking asylum. As many of these came from high TB incidence countries and were diagnosed with active TB at or shortly after entry in the country, TB incidence in Germany increased markedly [25]. We assume that such a strong epidemiological change would distort any seasonality in TB notifications.

As several studies found that seasonality varied by disease site [16, 17], we carried out additional time series analyses for pulmonary TB (also further stratified per mode of case finding) and extra-pulmonary TB separately. Further stratification of the extra-pulmonary per affected organ was not possible due to the low case numbers in the subgroups. Data were also analyzed separately for the following subgroups: men, women, and age groups 0–14 years, 15–65 years and 66+ years. Other factors that could influence seasonality, such as immunosuppressive conditions, could not be taken into account, as these are not part of the data set.

### Time series analysis

We analysed the monthly counts based on start of treatment and created integer-valued generalised autoregressive conditional heteroscedasticity (INGARCH) models using the” tscount “package [28] in R [29] where the conditional mean of the process is linked to its own previous values, to past observations and to potential covariates [28]. Making use of the well-known general framework of generalized linear model (GLM), the above model allows for a specification of the conditional distribution of the present counts through either poisson or negative binominal distributions using an identity or logarithmic link function [28]. INGARCH models are a particular type of integer-values time series specifications which have proven to be a sufficient alternative for classical real-valued time series models such as the AR (autoregressive), MA (moving-average) or ARMA (autoregressive–moving-average) specification when having integer-valued outcomes (counts) under study [30]_._ Another important class of models for time series with count data, such as INARMA (integer autoregressive moving average) models, makes use of the “thinning operator” to adapt the ARMA recursion to the integer-valued case. Compared to these, GLM-based models have the advantage that they describe covariate effects and negative correlations in a straightforward way and that a rich toolkit for this class of models is available [28]. Alternatively, “state space models” for counts can be used in time series analysis, which additionally allow to describe even more flexible data generating processes than GLM models. However, this often involves a more complicated model specification, while GLM-based models allow for predictions in a convenient matter due to their explicit formulation [28].

First, we fitted a model for trend. Different distributions for count data were considered and compared using the Akaike information criterion (AIC), the probability integral transform (PIT) and the cumulative periodogram of Pearson residuals, all of which indicated a clear preference for a negative binomial model distribution. Next, to investigate potential seasonality, we calculated the ordinary and partial autocorrelation functions (henceforth ACF and PACF) of the residuals aiming to assess whether these showed significant peaks (exceeding the 95% confidence interval) at yearly unit lags and whether a suspension bridge-like pattern, typical in the ACF of seasonal time series [16], could be identified. As the next step, we implemented the best fitting negative binomial INGARCH model including a seasonal component in the form of the twelfth order autocorrelation coefficient, corresponding to the regression on values of the conditional mean 12 units back in time. We then compared this model to the best fitting non-seasonal model based on AIC differences. The model with seasonal component was regarded to be a better fit when it had an AIC of at least 11 less than the model without seasonal component, as a difference in AIC of about 9–11 gives relatively little support for the model with the higher AIC [31]. Lastly, when the results of these analyses were not conclusive, or to confirm a suspected seasonality, we inspected the periodograms of the original time series, to identify important frequencies within the series. In the case of seasonality, we would expect a clear peak in the periodogram at a frequency of 0.0833, corresponding to a cycle of 1/0:0833 = 12 months [32].

## Results

### Study population

**Fig. 1:**
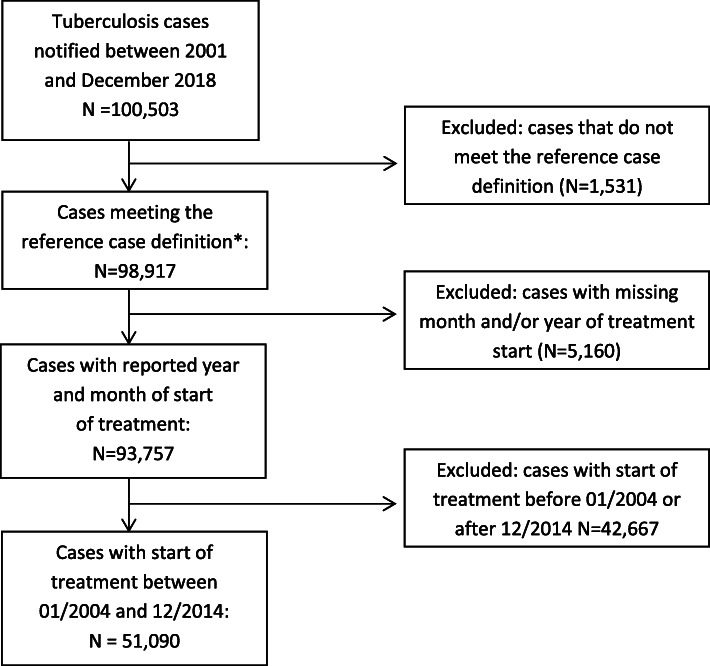
Flow chart of the in the time series analysis included and excluded TB cases in Germany with start of treatment between 2004 and 2014. * reference case definition: clinically diagnosed disease, clinically−/epidemiologically confirmed disease and clinically−/laboratory confirmed disease

The final dataset consisted of 51,090 TB cases (see Fig. 1). Pulmonary TB accounted for most of the cases with 78% (*N* = 39,714), while 22% of the notifications were extra-pulmonary TB cases (*N* = 11,219). Most of the pulmonary TB cases (*N* = 29,682, 84%) had been found through passive case finding (diagnosis after a patient presents himself with symptoms to a health worker), while a minority of 5742 pulmonary TB cases (16%) had been found through screening. Of all notified cases, 75% (*N* = 38,284) had laboratory confirmation, 60% (*N* = 30,788) were men and the median age was 48 years. The main demographic and clinical characteristics of the included cases are depicted in Table 1.

**Table 1:** Main demographic and clinical characteristics of the tuberculosis cases included in time series analysis 2004–2014 in Germany (*n* = 51,090)

Characteristics	Subgroups	*N*	%
Site of disease (*N* = 50,933)	Pulmonary TB	39,714	78
	Extra-pulmonary TB	11,219	22
Case finding (pulmonary TB) (*N* = 35,482)	Active case finding (screening)	5742	16
	Passive case finding	29,682	84
	Post-mortem	58	0,1
Diagnosis (*N* = 51,090)	Clinically diagnosed	11,672	23
	Clinically−/epidemiologically confirmed	1134	2
	Clinically−/laboratory confirmed	38,284	75
Sex (*N* = 51,009)	Female	20,221	40
	Male	30,788	60
Age (Years) (*N* = 51,083)	0–14	1890	4
	15–65	35,749	70
	66+	13,444	26

### Time series analysis

**Fig. 2:**
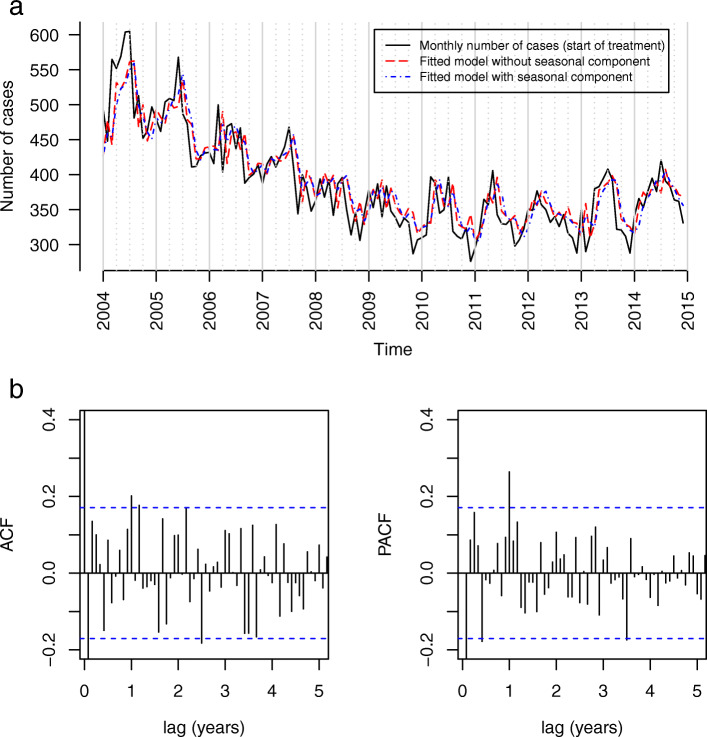
Notified tuberculosis cases in Germany with start of treatment between 2004 and 2014 (*N* = 51,090). **a** Monthly case numbers with the best fitting models with and without seasonal component. **b** ACF and PACF plots of residuals (model without seasonal component)

Figure 2a shows the monthly counts of all TB cases included in this study, aggregated based on date of start of treatment. The PACF plot of the residuals showed a significant peak (outside the 95% CI, depicted by the dotted lines) at the first yearly lag (see Fig. 2b). Inspecting the ACF plot, however, we did not observe an increase in the autocorrelation at several yearly lags, but at month 12, 14 and 26. The plot did not show a clear suspension bridge-like pattern, which would suggest the presence of a seasonal pattern, either. Furthermore, we did not find a clearly better model with a seasonal component (AIC difference 10.6, see Supplementary Table 1, Additional file 1). Lastly, the periodogram of the time series did not show a clear peak (See Supplementary Fig. 8, Additional file 1). These findings combined do not conclusively point towards a strong distinctly detectable seasonality.

**Fig. 3:**
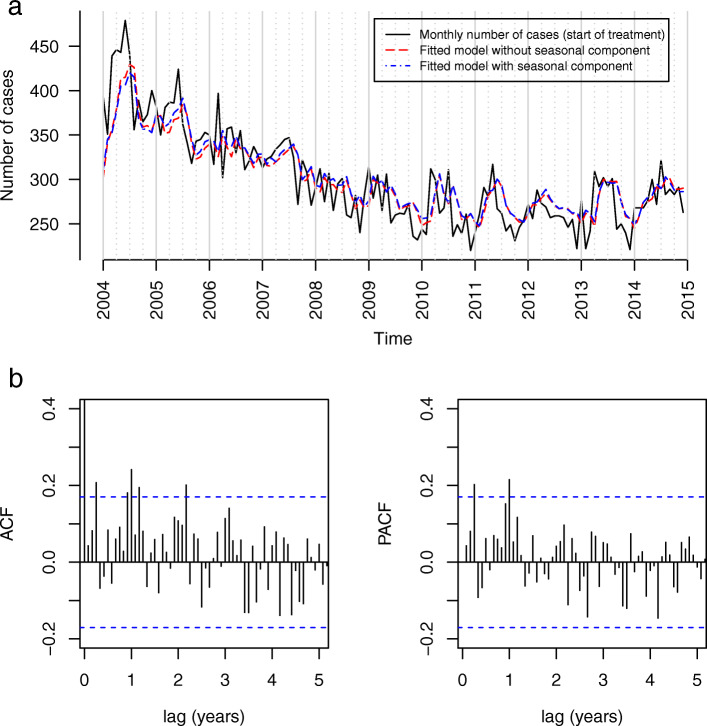
Notified pulmonary tuberculosis cases in Germany with start of treatment between 2004 and 2014 (*N* = 39,714). **a** Monthly case numbers with the best fitting models with and without seasonal component. **b** ACF and PACF plots of residuals (model without seasonal component)

The monthly counts of notifications of pulmonary TB only are shown in Fig. 3a. The ACF plot showed several significant peaks at the monthly lags 3, 11, 12, 14 and 26, and the PACF plot at lags 3 and 12 (see Fig. 3b), which does not point towards a clear seasonal pattern. The absence of a strong suspension bridge-like pattern in the ACF confirms the absence of seasonality. Furthermore, a clearly better fitting model with seasonal component could not be built (AIC difference 2.2) and the periodogram did not show a clear peak (See Supplementary Fig. 9, Additional file 1). To further investigate this absence of a distinct seasonality we analyzed the subgroup of pulmonary TB notifications stratified per mode of case finding: screening and passive case finding (diagnosis after a patient presents himself with symptoms to a health worker). However, we did not see a difference between the subgroups, as for neither a strong seasonality could be detected (see section 1 of Additional file 1).

**Fig. 4:**
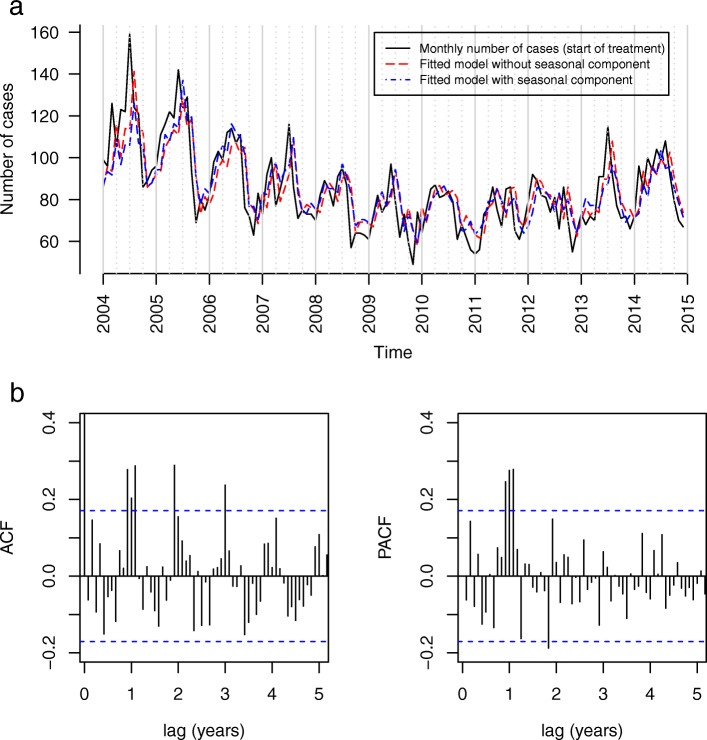
Notified extra-pulmonary tuberculosis cases in Germany with start of treatment between 2004 and 2014 (*N* = 11,219). **a** Monthly case numbers with the best fitting models with and without seasonal component. **b** ACF and PACF plots of residuals (model without seasonal component)

In the subgroup of extra-pulmonary cases (See Fig. 4a) we found seasonality with the ACF plot showing increasing autocorrelation at several yearly lags and a suspension bridge-like pattern (see Fig. 4b). The PACF showed significant peaks at month 11, 12 and 13, and the addition of a seasonal component clearly improved the model fit (AIC difference: 12.3). The periodogram showed a clear peak just before the frequency of 0.1, likely corresponding to 12 months (see Supplementary Fig. 10, Additional file 1). Table 2 shows the months with the highest and lowest numbers of notifications of extra-pulmonary TB for each year. The seasonal increase of extra-pulmonary TB notifications often peaked in late spring/summer (9 out of 11 years) and the troughs occurred in fall/early winter for all years.

**Table 2:** Peak months and trough months per year for notified extra-pulmonary tuberculosis cases in Germany with treatment start between 2004 and 2014

Year	Peak month	Trough month
2004	July	October
2005	June	October
2006	June	November
2007	July	September
2008	July	September
2009	June	November
2010	April	December
2011	May/September	January
2012	February	November
2013	July	December
2014	August	December

Additional analyses for men and women and for the different age groups separately revealed no clear difference in seasonality between the subgroups (see section 2 and 3 of Additional file 1).

## Discussion

We found seasonality in the extra-pulmonary TB cases notified in Germany between 2004 and 2014, with peaks in late spring/summer and troughs in fall/early winter, while no clear seasonality was found in pulmonary TB cases or in the set of all cases. This could point towards differences in disease progression between different clinical disease manifestations of TB. Such differences would be in line with the results of a study by Borgdorff et al. [33], who found a shorter incubation period for extra-pulmonary TB. We did not find any clear differences in seasonality between men and women nor between the different age groups.

The detected pattern of peaks and troughs in extra-pulmonary cases in our study is consistent with the pattern detected in other studies for TB in different countries [1–19]. One of the explanations mentioned in these studies is increased transmission in winter due to more time being spent indoors. However, the long, variable incubation times in extra-pulmonary TB make winter indoor crowding an unlikely factor behind the seasonal peak in spring/summer for this subgroup [17]. Other possible explanations would be deficiency of Vitamin D and general impaired immunity in winter, presumably leading to increased numbers of TB disease onsets in winter, followed by peak of diagnosis and corresponding treatment start in spring/summer. These and other possible mechanisms behind the detected seasonality would be a relevant topic for additional studies, as this was not the aim of our investigation. The proportion of notified extra-pulmonary TB cases in Germany and neighboring countries has increased in the last few years; therefore, this is a research topic of increasing relevance.

Our findings for Germany are consistent with two other studies in the Netherlands which detected similar seasonality only/primarily in extra-pulmonary cases [16, 17]. We cannot, however, exclude that for pulmonary TB the seasonality might be masked and thus more difficult to detect. Therefore, we tested the hypothesis that seasonality in pulmonary TB notifications is obscured by cases found through screening activities, analyzing seasonality for pulmonary TB cases stratified by mode of case finding. Our results did not show a difference in the detection of seasonality between the cases found through screening and those found through passive case finding: for neither subgroup separately a clear seasonality could be detected. A second hypothesis would be that in winter health care workers might more often attribute symptoms of pulmonary TB to other respiratory illnesses [5]. Such decreased awareness could lead to longer diagnostic and treatment delays in winter and a shift of TB diagnosis to later times in the year.

Our study has several limitations. First, we used date of start of treatment as a proxy for the disease onset, as the latter is only notified for 42% of the cases. However, even when the disease onset is notified, the date itself contains uncertainty, as TB is generally a slowly progressive disease which likely makes it difficult to determine the exact date of disease onset. This may especially apply to extra-pulmonary TB, as it often has a non-specific clinical presentation, leading to a delay in diagnosis [34]. However, the systematic application of this definition on all cases might lead to a shift in the peak months, but otherwise should not affect the analysis of a seasonal pattern. Second, the monthly case numbers for children (between 0 and 14 years old) were too small to assess seasonality in this subgroup. Children are, however, an important subgroup for analyzing seasonality of TB, as in this subgroup disease likely reflects recent transmission rather than activation of latent TB [4]. Relatively low case numbers in this group and in the group of the elderly (see section 3 of Additional file 1) did not allow further stratification of these subgroups. For the same reason, further stratification of the extra-pulmonary cases per affected organ was not possible. Finally, our study did not control for potential impacts of exterior factors such as climate and weather conditions, or geographical region.

## Conclusion

We found a seasonal pattern with peaks in spring/summer and through in fall/early winter apparent in notifications of extra-pulmonary TB only. These results indicate that seasonality of disease onset might be depending on the disease site, possibly pointing towards differences in disease progression between the different clinical disease manifestations of TB. Sex appears not to be a driver of seasonality, and the role of age remains unclear as this could not be sufficiently investigated due to low case numbers for children.

## Supplementary Information

**Additional file 1.** Results of analyses of: Pulmonary TB notifications stratified per mode of case finding (Section 1), TB notifications stratified by sex (Section 2), TB notifications stratified per age group (Section 3). AIC values for the fitted models (Section 4). Periodograms for the original time series (Section 5).

## Data Availability

All data utilized in the study were collected in accordance with the German ‘Protection against Infection Act’ (‘Infektionsschutzgesetz’), which has very stringent data protection guidelines. The Data Protection Office of the Robert Koch Institute restricts sharing of any case-based surveillance data externally as the data are only pseudo-anonymized and contain potentially identifying patient information. However, an anonymous data set can be made available under reasonable request from the corresponding author, with permission from the Research Data Management Unit of the Robert Koch Institute.

## Abbreviations

ACF: Autocorrelation functions
AIC: Akaike information criterion
AR: Autoregressive
ARMA: Autoregressive–moving-average
GLM: Generalized linear model
INARMA: Integer autoregressive moving average
INGARCH: Integer-valued generalised autoregressive conditional heteroscedasticity
MA: Moving-average
PACF: Partial autocorrelation function
PIT: Probability integral transform
TB: Tuberculosis
