# Supplementary material for: Seasonality in extra-pulmonary tuberculosis notifications in Germany 2004-2014- a time series analysis
Source: BMC Public Health. 2021 Apr 6;21:661. doi: 10.1186/s12889-021-10655-6 (PMC8025493; doi:10.1186/s12889-021-10655-6)
Supplement: Supplementary file 1 — Additional file 1. Results of analyses of: Pulmonary TB notifications stratified per mode of case finding (Section 1), TB notifications stratified by sex (Section 2), TB notifications stratified per age group (Section 3). AIC values for the fitted models (Section 4). Periodograms for the original time series (Section 5). [file 12889_2021_10655_MOESM1_ESM.pdf]

Additional file 1

Section 1: Pulmonary TB notifications stratified per mode of case finding

The monthly notifications of pulmonary TB cases which were found through screening are shown in Supplementary Figure 1.The autocorrelation plots show no seasonality with no significant peaks at yearly lags, nor could a better fitting model with seasonal component be built (see Supplementary Table 1). However, the best fitting models might not describe the data well enough to be used for assessing seasonality; possibly due to the relatively low monthly case numbers. For the pulmonary TB cases found through passive case finding (Supplementary Figure 2) both the ACF and the PACF show a significant peak at the 14 month lag only and no suspension bridge-like pattern could be detected. The model with seasonal component only barely fits the data better than the one without (AIC difference: 6.5).

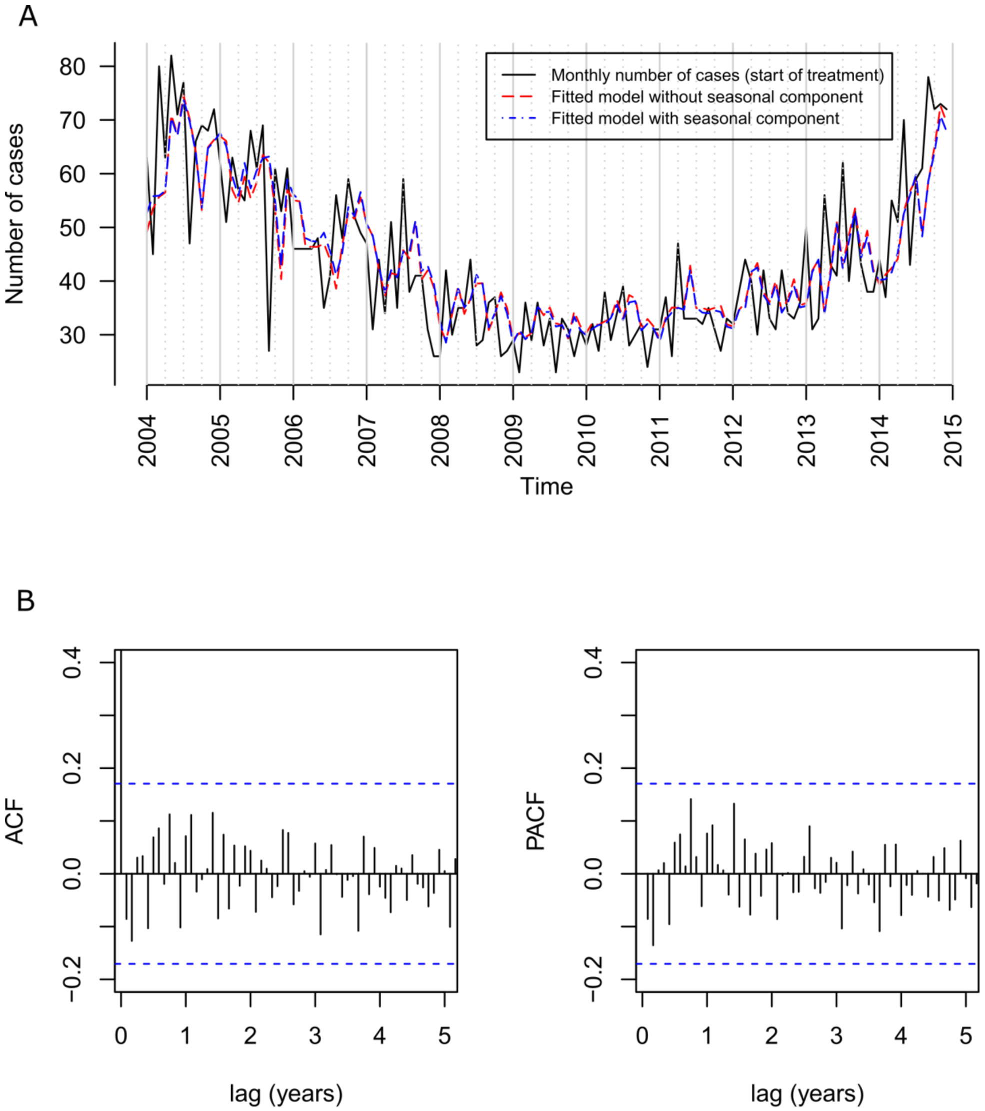

Supplementary Figure 1: Notified pulmonary tuberculosis cases in Germany found through screening, with start of treatment between 2004 and 2014 (N=5,742). A) Monthly case numbers with the best fitting models with and without seasonal component. B) ACF and PACF plots of residuals (model without seasonal component).

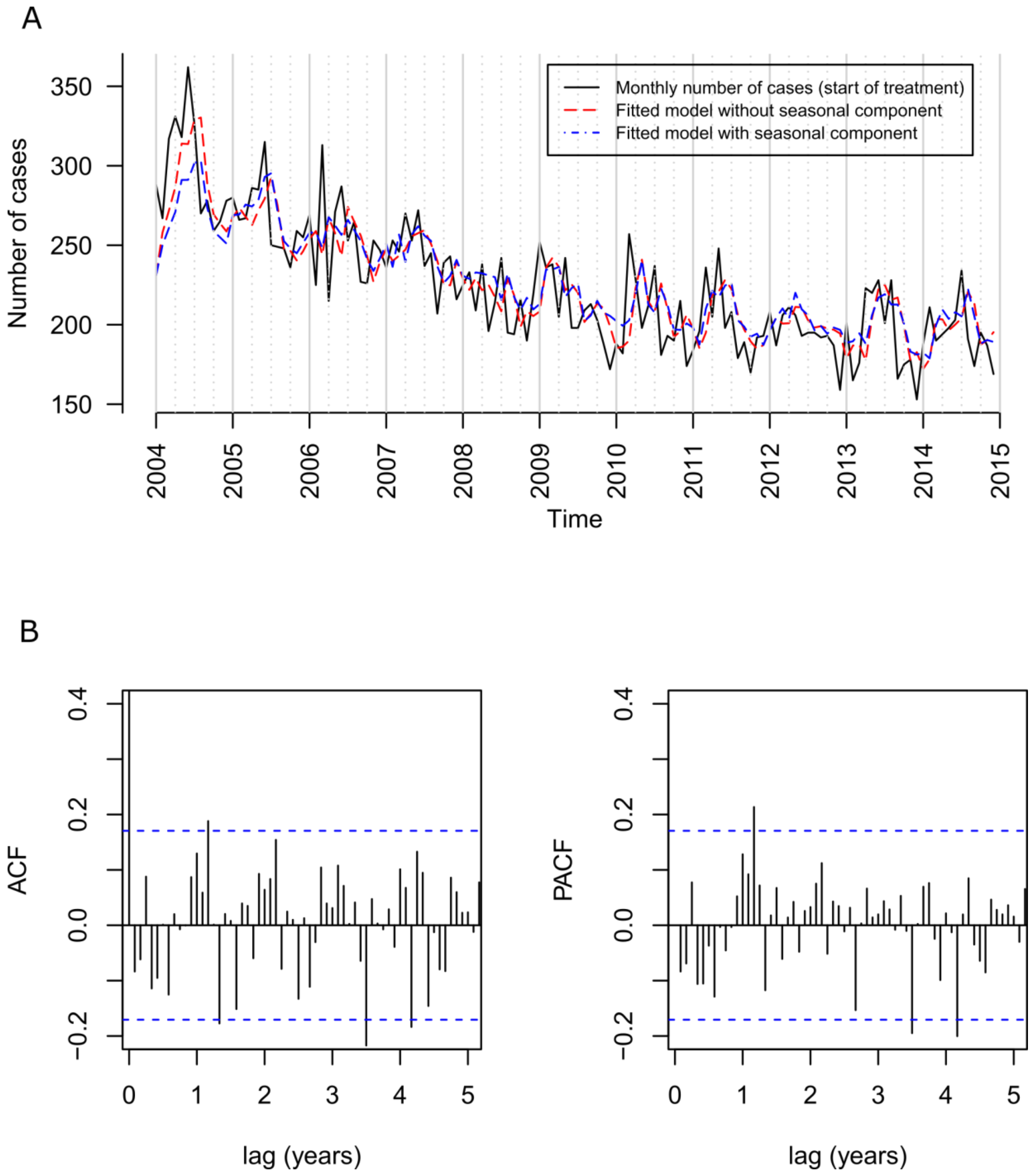

Supplementary Figure 2. Notified pulmonary tuberculosis cases in Germany found through passive case finding, with start of treatment between 2004 and 2014 (N=29,682). A) Monthly case numbers with the best fitting models with and without seasonal component. B) ACF and PACF plots of residuals (model without seasonal component).

Section 2: TB notifications stratified by sex

The autocorrelation plots for women and men separately did not show a strong seasonality (see Supplementary Figures 3 and 4) and for neither subgroup a clearly better fitting seasonal model could be built (see Supplementary Table 1).

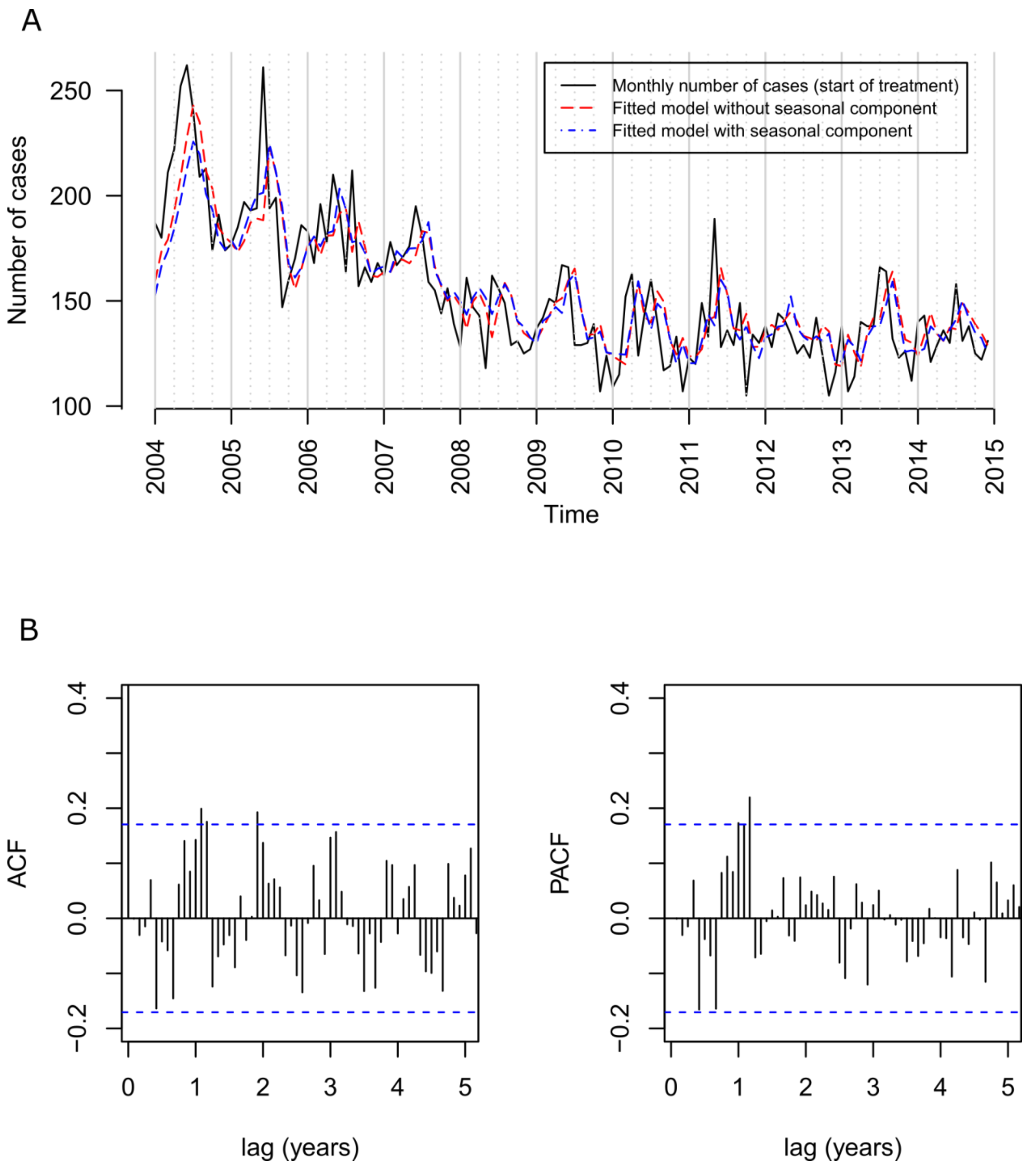

Supplementary Figure 3. Notified tuberculosis cases in women in Germany with start of treatment between 2004 and 2014 (N=20,221). A) Monthly case numbers with the best fitting models with and without seasonal component. B) ACF and PACF plots of residuals (model without seasonal component).

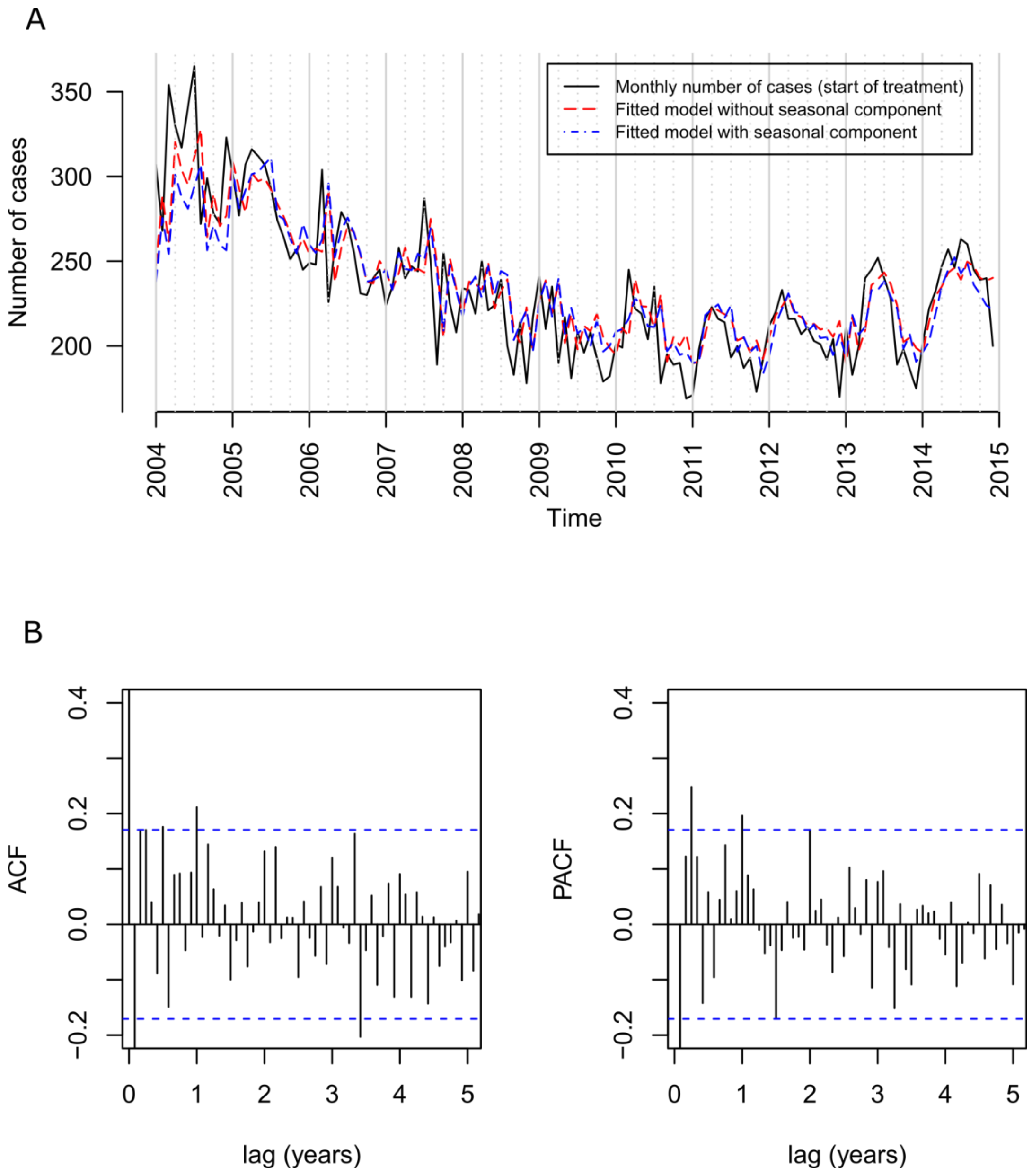

Supplementary Figure 4. Notified tuberculosis cases in men in Germany with start of treatment between 2004 and 2014 (N=30,788). A) Monthly case numbers with the best fitting models with and without seasonal component. B) ACF and PACF plots of residuals (model without seasonal component).

Section 3: TB notifications stratified per age group

Due to the low number of cases per month (min. 4, max. 28), in the age group 0-14 years (Supplementary Figure 5) no appropriate model could be fitted for this subgroup and therefore the seasonality was not assessed. For the age groups 15-65 years and 66+ years the autocorrelation plots showed no clear seasonality (see Supplementary Figure 6 and 7) and no clearly better fitting models with seasonal component could be built (see Supplementary Table 1).

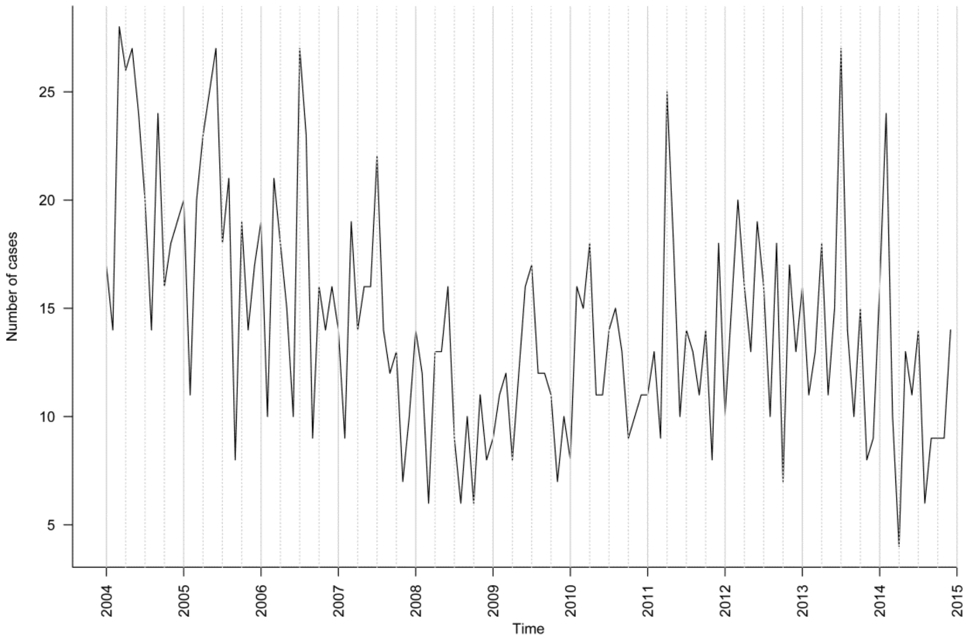

Supplementary Figure 5. Monthly numbers of notified tuberculosis cases in children (younger than 15 years old) in Germany with start of treatment between 2004 and 2014 (N=1,890).

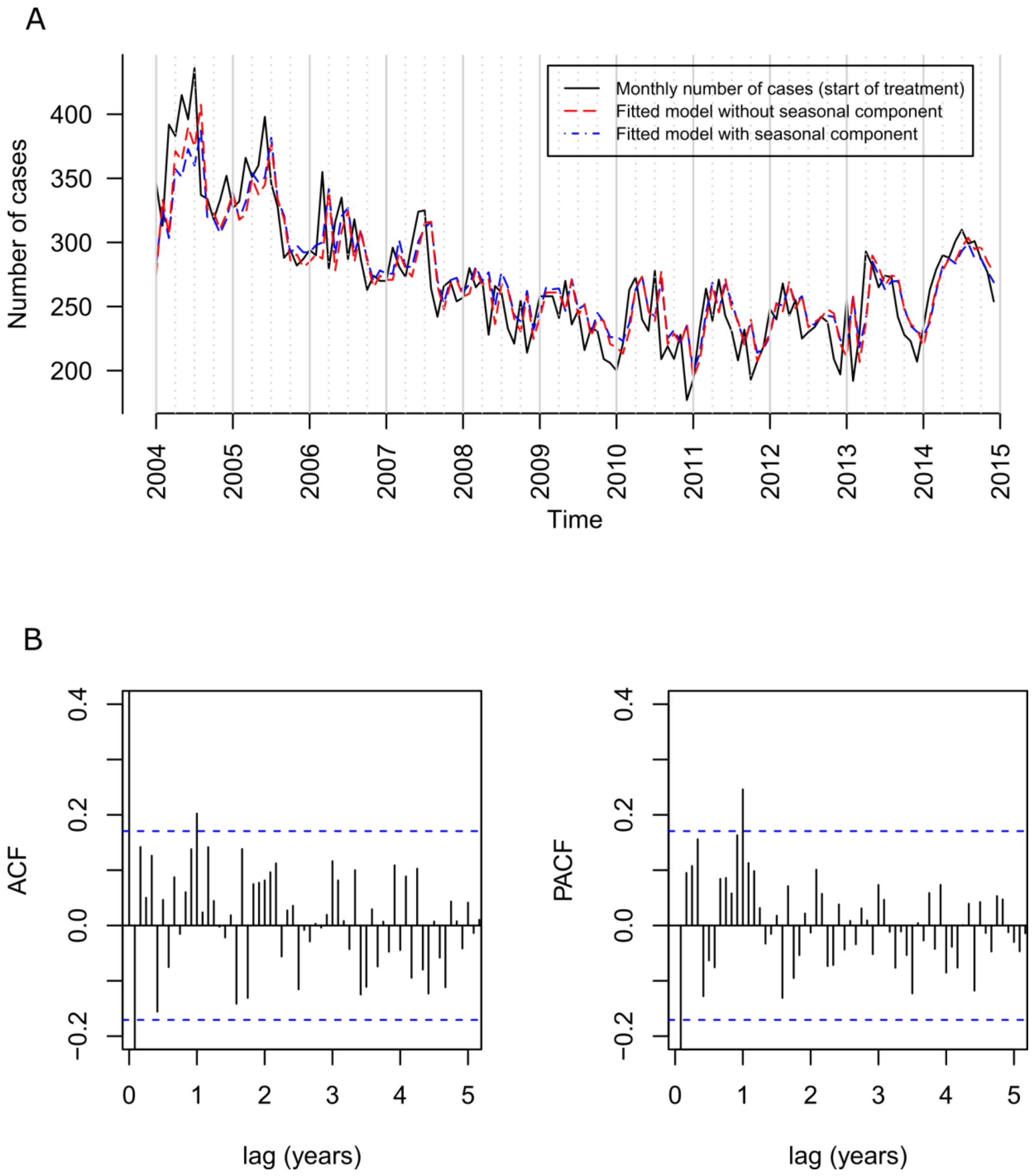

Supplementary Figure 6. Notified tuberculosis cases in people aged 15-65 years in Germany with start of treatment between 2004 and 2014 (N=35,749). A) Monthly case numbers with the best fitting models with and without seasonal component. B) ACF and PACF plots of residuals (model without seasonal component).

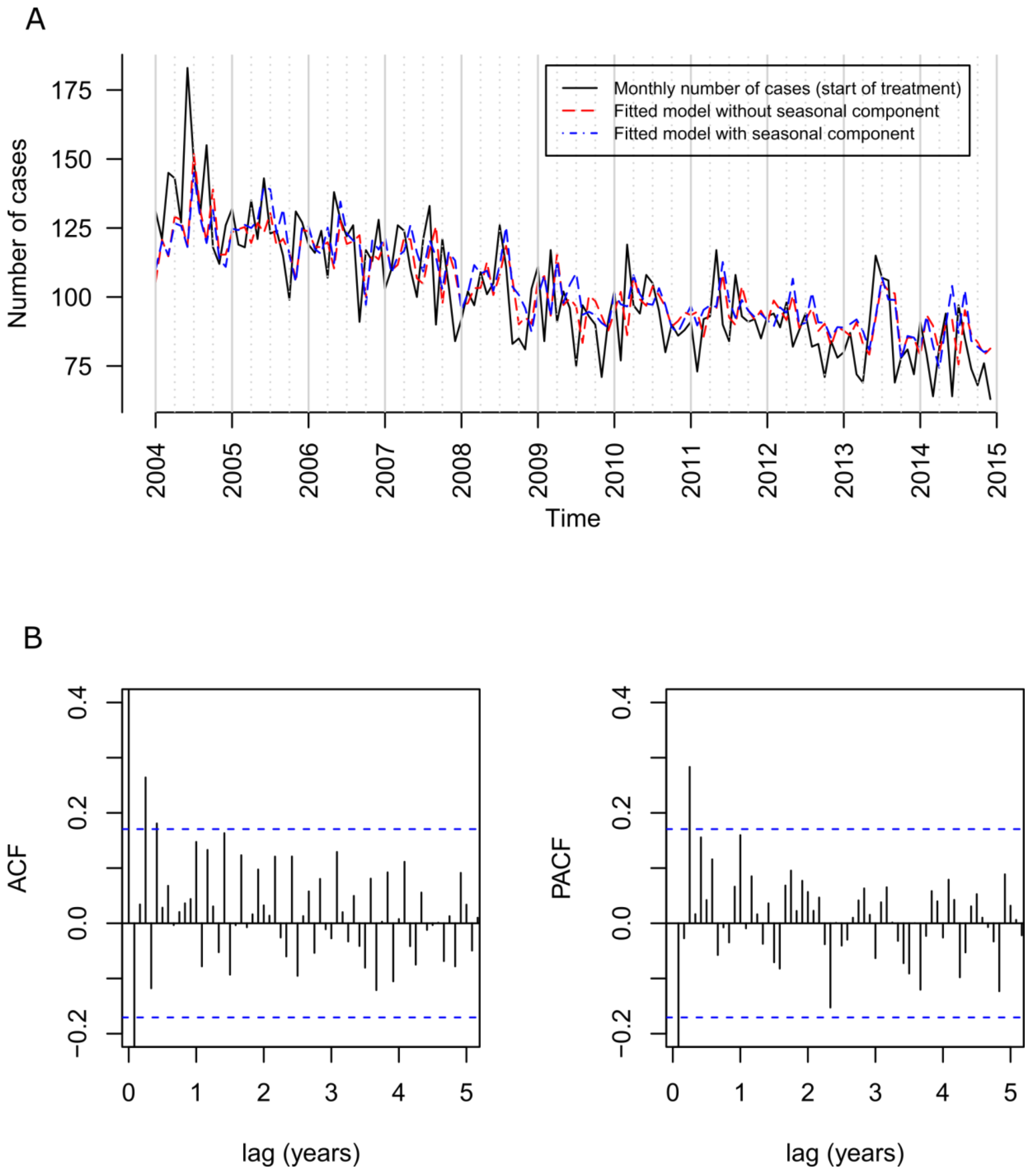

Supplementary Figure 7. Notified tuberculosis cases in people older than 65 years in Germany with start of treatment between 2004 and 2014 (N=13,444). A) Monthly case numbers with the best fitting models with and without seasonal component. B) ACF and PACF plots of residuals (model without seasonal component).

Section 4: AIC values for the fitted models

Subgroups of TB notifications	AIC of model without seasonal component	AIC of model with seasonal component	AIC difference*
**All notifications**	1316.537	1305.940	10.6
**Pulmonary TB**	1265.229	1263.017	2.2
**Screening**	950.2784	949.4594	0.8
**Passive case finding**	1222.957	1216.448	6.5
**Extra-pulmonary TB**	1044.358	1032.009	**12.3**
**Women**	1152.050	1157.112	- 5.1
**Men**	1219.882	1217.334	2.5
**People aged 15-65 years**	1256.024	1249.918	6.1
**People older than 65 years**	1098.516	1096.411	2.1

* a positive difference of at least 11 points towards seasonality

Supplementary Table 1. The AIC values for the fitted models (model without and model with seasonal component) and the difference in AIC per subgroup.

Section 5: Periodograms for the original time series

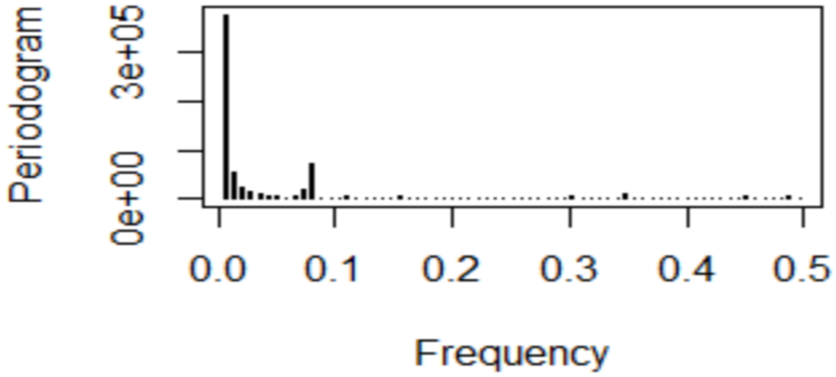

Supplementary Figure 8. Periodogram of the time series for all notified tuberculosis cases in Germany with start of treatment between 2004 and 2014 (N=51,090)

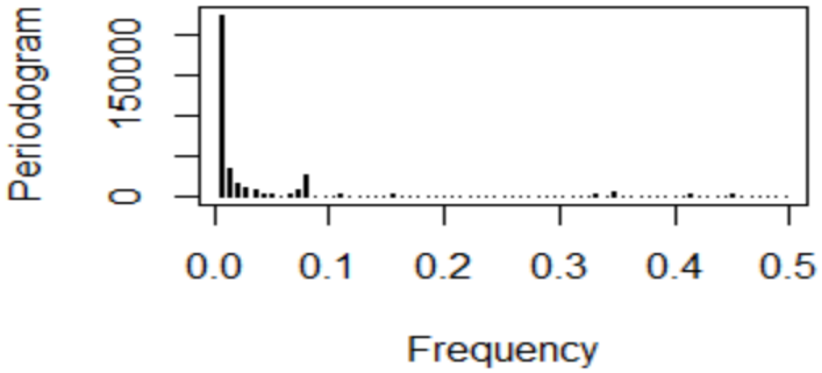

Supplementary Figure 9. Periodogram of the time series for notified pulmonary tuberculosis cases in Germany with start of treatment between 2004 and 2014 (N=39,714)

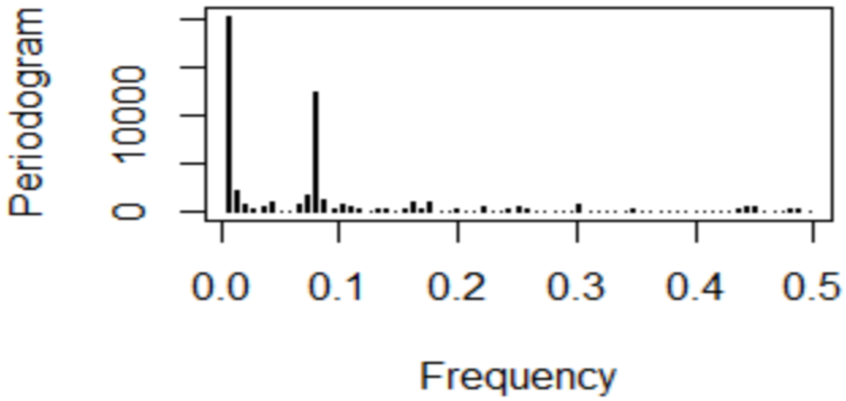

Supplementary Figure 10. Periodogram of the time series for notified extra-pulmonary tuberculosis cases in Germany with start of treatment between 2004 and 2014 (N=11,219)
